# Recent Developments in the Study of Plant Microbiomes

**DOI:** 10.3390/microorganisms9071533

**Published:** 2021-07-19

**Authors:** Bernard R. Glick, Elisa Gamalero

**Affiliations:** 1 Department of Biology, University of Waterloo, Waterloo, ON N2L 3G1, Canada; glick@uwaterloo.ca; 2 Dipartimento di Scienze e Innovazione Tecnologica, Università del Piemonte Orientale “A. Avogadro”, Viale Teresa Michel, 11, 15121 Alessandria, Italy

**Keywords:** soil bacteria, plant growth-promoting bacteria, PGPB, seed microbiomes, root microbiomes, organic agriculture

## Abstract

To date, an understanding of how plant growth-promoting bacteria facilitate plant growth has been primarily based on studies of individual bacteria interacting with plants under different conditions. More recently, it has become clear that specific soil microorganisms interact with one another in consortia with the collective being responsible for the positive effects on plant growth. Different plants attract different cross-sections of the bacteria and fungi in the soil, initially based on the composition of the unique root exudates from each plant. Thus, plants mostly attract those microorganisms that are beneficial to plants and exclude those that are potentially pathogenic. Beneficial bacterial consortia not only help to promote plant growth, these consortia also protect plants from a wide range of direct and indirect environmental stresses. Moreover, it is currently possible to engineer plant seeds to contain desired bacterial strains and thereby benefit the next generation of plants. In this way, it may no longer be necessary to deliver beneficial microbiota to each individual growing plant. As we develop a better understanding of beneficial bacterial microbiomes, it may become possible to develop synthetic microbiomes where compatible bacteria work together to facilitate plant growth under a wide range of natural conditions.

## 1. Introduction

An enormous number of bacteria are typically found in soil. Various soils contain ~1 × 10^6^ to 1 × 10^9^ bacterial cells per gram of soil, often including as many as 1 × 10^6^ different taxa [1]. These bacteria may be beneficial (plant growth-promoting bacteria; PGPB), harmful (phytopathogens) or neutral in terms of their interaction with plants [2]. The greatest number of bacteria are typically found in the plant rhizosphere i.e., the region of the soil immediately around the roots [3]. The high concentration of bacteria occurring around plant roots is a direct consequence of the fact that plant roots commonly exude a significant fraction of the carbon that is fixed through photosynthesis [4,5]. In addition to being present in the rhizosphere, PGPB may also be endophytic, i.e., some bacteria are able to colonize the plant’s interior, symbiotic, i.e., some bacteria colonize the interior of the roots of specific plants by forming nodules on the plant root, or phyllospheric (i.e., they are found on the surfaces of plant leaves and stems) [2].

**Figure 1:**
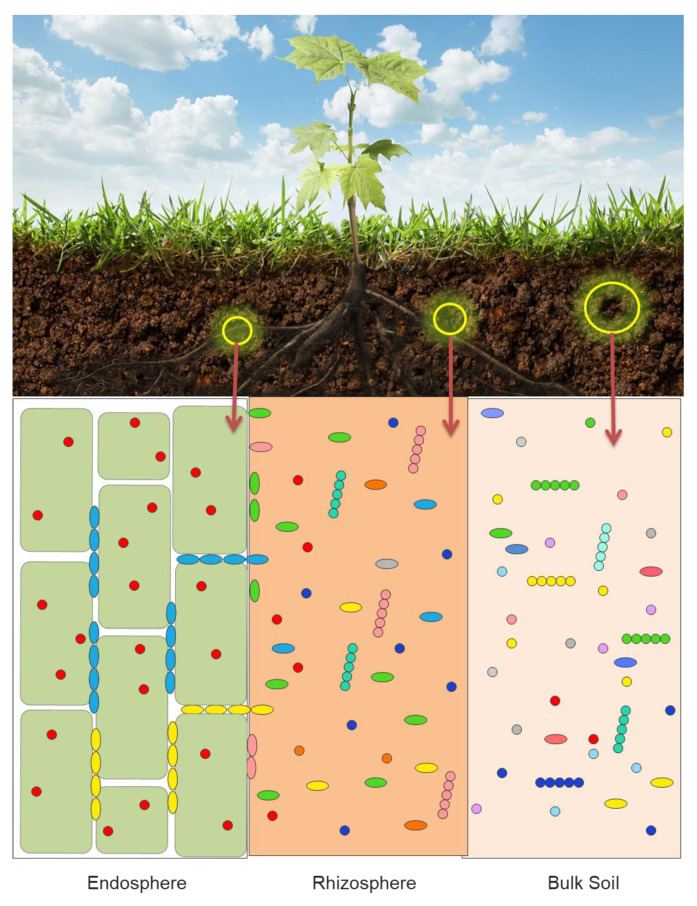
Schematic representation of some of the microorganisms contained within the plant root endosphere, rhizosphere and bulk soil.

The vast majority of the reported laboratory and greenhouse studies of the interaction between soil bacteria and plants have been focused on the mechanisms used by individual bacterial strains, either PGPB or pathogens. However, in the past 10–15 years many scientists have turned their attention to the functioning of groups of bacteria, that often act together to affect plant growth and development. Much of the available evidence that exists to date indicates that different plants attract different cross-sections of the soil bacteria [1,6,7,8,9,10,11]. Each plant exudes or secretes through its roots a unique mixture of small molecules that attracts a specific fraction of the soil bacteria. In addition, different bacteria are attracted to and found in the plant microbiota (the characteristic microbial community occupying different parts of a plant). Thus, it is shown schematically in Figure 1 that microbiota that is found in a plant rhizosphere is quite different from the microbiota within the plant root tissues (the endosphere) and from the biota found in the bulk soil.

While plant microbiomes may include bacteria, fungi, oomycetes and archaea, the majority of the studies published on this topic deal with bacteria (bacteriome), and to a lesser extent, fungi (mycobiome). Consequently, this review is directed toward developing an understanding of the functioning of bacteria within plant microbiomes. Although the microbiome of the phyllosphere impact plant health and, often, food production, only a limited number of studies have been aimed at discovering these particular communities, well adapted to the hostile leaf environment and mainly dominated by Alphaproteobacteria and by the genera *Methylobacterium* and *Sphingomonas* [12]. Given that the plant root is the major site where plants and bacteria interact with one another, it is not surprising that the vast majority of soil bacteria are members of the root microbiota (Figure 1). Root exudates are generally unique to each plant. As a consequence, different plants have a propensity for attracting specific subsets of soil bacteria. It is generally thought that the chemical composition of plant root exudates is involved in recruiting or selecting from the bulk soil the bacteria that make up a plant’s root microbiome [6,13,14,15,16,17]. However, the rhizosphere microbiota is only one of the key determinants of plant yield [18]. In addition to specific root exudates (a direct consequence of the plant host genotype), root microbiomes are also dependent upon soil type and environmental changes [19]. Oddly enough, different cultivars (subspecies) of the same plant can sometimes produce different panels of metabolites that make up their root exudates and therefore select for different root microbiomes. While plant-derived carbon appears to play a key role in determining the bacterial composition of the rhizosphere microbiome, soil-derived carbon compounds also can have an impact on the composition of rhizosphere microbiome [20]. Of the bacterial inhabitants of the plant rhizosphere, a small number of those bacteria are able to enter into the root tissues and subsequently permanently colonize the plant’s endosphere, i.e., they become the basis of an endophytic microbiome.

The soil and even the rhizosphere contain a wide variety of microorganisms including those that are beneficial and those that are potentially pathogenic. This being the case, it is essential to ask how plants recruit beneficial microorganisms, while for the most part, restrict (or try to restrict) pathogens. As indicated by Thoms et al. [21], “Once microbes are present, plants must decide to tolerate their presence, engage in mutualistic symbiosis, or mount an immune response”. These researchers have suggested that there are three stages in the interaction between plants and microorganisms. These include (i) metabolic gating where the production of specific metabolites by the plant restricts microbial access to the plant; (ii) dual receptor recognition in which specific signals from the plant and the microorganism bind to one another’s receptors and initiate either increased interaction or immunity; and (iii) integration of environmental signals with immune homeostasis. In metabolic gating, plants synthesize nutrients that only some microbes can use, or they can produce antimicrobial compounds that are toxic to only some microbes. Plant roots exude compounds using a variety of (mostly passive) mechanisms including diffusion, ion channels and vesicle transport. In general, ABC transporters are responsible for exudation of lipids and flavonoids, anion channels for sugars and other carbohydrates, metal transporters for various metals, aquaporins for water and uncharged molecules, and vesicles for high-molecular-weight compounds [4,5,22]. In dual receptor recognition, the exchange of signals combined with plant and microbial genetic potential helps to determine the ultimate fate of the interaction [23]. Finally, although microbes in the soil exist in the presence of a large number of other microbes, plants have to integrate their nutritional status with their expression of immunity to maintain homeostasis [21,24].

In the past few years there has been a veritable explosion of studies directed toward understanding the nature of plant microbiomes (Table 1). This enormous work is based on both scientists’ ability to rapidly characterize large numbers of bacteria and on the precedents set in the developing understanding and importance of human–bacterial microbiomes. To characterize the bacteria, soil and other particulate matter are removed from the bacteria before genomic DNA is extracted and 16S rDNA is PCR amplified, used to prepare a DNA library and then sequenced. In some of the studies mentioned in this review, fungal rDNA was also characterized. This notwithstanding, the focus of this review is the behavior of the bacterial microbiomes.

## 2. Soil Bacteria and Plant Growth

**Figure 2:**
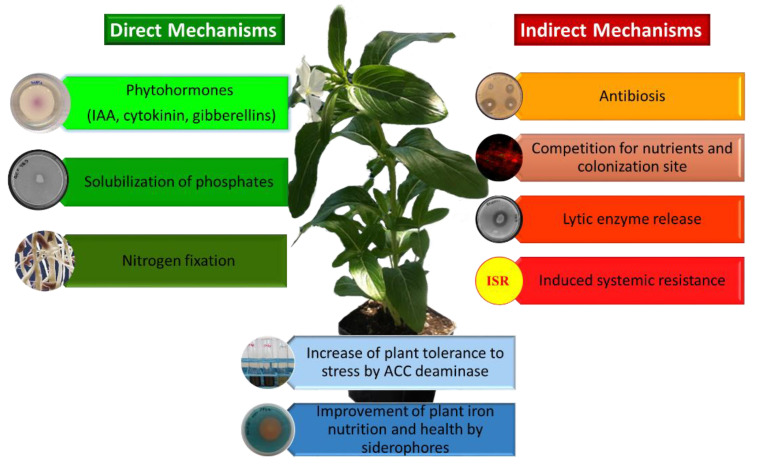
Mechanisms used by PGPB to improve plant growth and development.

PGPB can benefit plant growth and development in a number of different ways and environmental conditions [2,80,81,82,83,84]. These include facilitating plant growth by increasing plant biomass, increasing plant mineral content (iron, phosphorus, potassium), providing plants with fixed nitrogen, increasing root and/or shoot length, enhancing seed germination, protecting plants from a wide range of phytopathogenic organisms (i.e., biocontrol), increasing plant tolerance to a wide range of environmental stresses (e.g., salt, flooding, drought, extremes of temperature, organic and inorganic soil contaminants), increasing the production of useful secondary metabolites, and increasing the overall level of plant nutrition. Conceptually, PGPB may facilitate plant growth both directly and indirectly [84]. Direct promotion of plant growth occurs when a PGPB either facilitates the acquisition of a required nutrient from the environment or adjusts and optimizes the level of hormones within a plant. As a consequence of the direct mechanisms, plants colonized by PGPB, show an increased plant biomass, yield and an improved nutritional value of seeds and fruits [85]. On the other hand, indirect promotion of plant growth reflects the ability of a PGPB to decrease the deleterious effects of phytopathogens on plants. Direct promotion of plant growth and development by PGPB may occur by producing auxin (most notably indoleacetic acid; IAA), ACC (1-aminocyclopropane-1-carboxylate) deaminase, cytokinin, or gibberellin, fixing atmospheric nitrogen, or solubilizing environmental phosphorus, iron or potassium. The indirect mechanisms used by PGPB to promote plant growth include ACC deaminase lowering of stress ethylene levels, synthesis of pathogen-inhibiting antibiotics, synthesis of pathogen fungal cell wall-degrading enzymes, outcompeting pathogens for nutrients (including for available iron) and/or space, synthesis of fungal pathogen-inhibiting hydrogen cyanide, and induction of systemic resistance mechanisms (ISR) [2,84] (Figure 2). 

All these plant beneficial traits are at the base of the procedure for the selection of PGPB consisting in: (i) isolation of microorganisms from soil, rhizosphere or endosphere, (ii) characterization of the bacterial physiological activities, (iii) determination of the impact of the PGPB on the growth of plants under controlled and greenhouse conditions, and under optimal and stressed environmental conditions, (iv) assessment of the PGPB ecological safety, (v) development of a formulation satisfying the farmer’s needs and ensuring the bacterial viability, and (vi) marketing and registration. However, the occurrence of physiological plant beneficial strains is usually performed by qualitative or quantitative in-vitro tests, whose results do not always reflect the real PGPB performance in an open field [86]. Moreover, not all PGPB utilize the same mechanisms to facilitate plant growth; each encodes only a few of the above-mentioned mechanisms that are beneficial to plants. No one PGPB strain ever contains all of these traits. This is because increasing the number of genes that are involved in facilitating plant growth is likely to cause an increased metabolic load on the PGPB, thereby making it less competitive with other soil bacteria in the environment [87]. With this consideration, it is easy to understand why a microbiome containing a range of different PGPB (each with its own plant beneficial trait) might be more effective in promoting plant growth in the environment than a single (more limited) PGPB strain. Moreover, different plants may respond differently to a particular PGPB strain depending upon the phenological stage and the health status of the plant. Finally, it is important to keep in mind that the bacterial component is not unique in the rhizosphere. Other beneficial microorganisms such as mycorrhizal fungi, establishing mutualistic symbiosis with 90% of the land plants, strictly interact with the plant bacteriome, often leading to synergistic effects on plant growth (for a recent review see [88]).

## 3. Microbiomes and Stress

Various stresses can have a significant effect on plant metabolism and hence, on the composition of root exudates. Plants that are grown in the field are subjected to a wide range of both biotic and abiotic stresses. The biotic stress factors include pathogenic fungi, bacteria, viruses, nematodes and insects, all of which may be significantly deleterious to plant growth and health. In addition, a number of abiotic stresses may also negatively affect plant growth; these include high and low temperature, high light levels, drought, flooding, high salt, toxic organic compounds, inhibitory metals and radiation. Any one of these stresses can have a significant negative effect on plant growth and development. Moreover, a plant may sometimes encounter several environmental stresses at the same time. Of course, plants have their own built in defenses against many types of environmental stresses, however, in many cases a plant’s defenses provide insufficient protection against this environmental onslaught. Fortunately, a large number of rhizosphere microbiota protect plants against a wide range of environmental stresses. The protection against a wide range of environmental stresses provide a simple rationale for understanding why plants actively select PGPB. In exchange for the many ways in which PGPB facilitate plant growth, the bountiful root exudates provide PGPB with a much needed food source.

Despite the fact that different plants respond in various ways to biotic and abiotic stresses, nearly all environmental stresses induce plants to synthesize an increased level of the phytohormone ethylene [89]. Milder stresses cause the synthesis of low levels of ethylene that, in turn, induce the activation of the expression of plant defensive genes creating a protective response to the environmental stress in the plant. More severe or prolonged stresses often cause the synthesis of high levels of ethylene within the plant leading to plant senescence, chlorosis and abscission, typically exacerbating the effects of the environmental stress. Thus, when plants are highly stressed, “a large portion of the damage that occurs to the plant is due to autocatalytic ethylene synthesis” and not merely from the direct action of the stress [90]. A partial remedy to the deleterious and complicating effects of increased levels of ethylene is to treat plants (prior to the onset of any stress) with bacteria that synthesize the enzyme ACC deaminase [84,91,92,93,94,95,96,97]. In fact, lowering plant ethylene levels using PGPB that synthesize ACC deaminase, is a highly effective strategy to decrease the damage to plants caused by fungal pathogens, nematodes, flooding, drought, high salt, environmental contaminants and a number of other environmental stresses.

Not surprisingly, biotic stress has typically been observed to alter the microbial communities that are associated with the stressed plant. (i) In *Verticillium* diseased cotton plants, the numbers of arbuscular mycorrhizal fungi and the plant beneficial bacteria decreased while the numbers of plant pathogenic fungi increased compared to healthy plants [45]. (ii) In examining the relationship between the soil microbiome and strawberry plant resistance to the pathogens *Verticillium dahliae* and *Macrophomina phaseolina*, the resistant plants microbiome had a higher abundance of known beneficial bacteria and biocontrol bacteria [50]. (iii) In pepper plants, it was observed that aphid infestation (which occurs in the leaves) altered root exudation which led to the plant recruiting rhizobacteria that decreased the resistance of the pepper plants to the aphids [98]. Thus, foliar insects modulated the bacterial microbiome and increased the susceptibility of the plant to the foliar insects. (iv) One study examined the effect of compost on the growth of tomato plants and the ability of this treatment to suppress disease caused by added *Fusarium oxysporum* f. sp. *lypersici* and *Verticillium dahlia* [15]. With these pathogens, the disease intensity was significantly decreased in the presence of the added compost (which altered the plant microbiome).

These limited number of experiments suggest that the presence of fungal pathogens (i.e., a biotic stress) alters the root exudates of plants and this change may favor the pathogenicity of the fungal pathogen. However, adding a suppressive compost, which is in effect adding a consortium of biocontrol bacteria, may overcome the negative outcome that would otherwise ensue from the presence of phytopathogens. It will be interesting to examine plant microbiomes following biotic stresses other than the presence of fungal pathogens to assess the nature of the changes to the plant microbiome. 

In recent years, plant abiotic stress and its amelioration has received quite a lot of attention [83,99,100,101,102,103,104,105,106]. By far, of the many abiotic stresses that inhibit plant growth the most attention has been paid to salt stress [107,108,109,110]. This is likely because of the fact that around half of the world’s agricultural land that is irrigated is adversely affected by salt. Because of insufficient water, many regions are under-irrigated causing salts to accumulate in the soil. 

Abiotic stresses such as drought and high salt have been shown to have a significant inhibitory effect on crop yields and the microbiomes of those crops. (i) In one three-year field study of wheat plants, significant differences between dryland and irrigated crops were observed. In this study, irrigation led to small increases in the overall diversity within the rhizosphere microbiome [48]. Thus, an adequate amount of water is needed for a healthy rhizosphere microbiome. (ii) A study of cotton plants grown in field soil under controlled conditions suggested that the cotton plants were able to access a bacterial community that improved its drought tolerance [46]. (iii) In another study, scientists showed that drought delays the development of the early sorghum root microbiome and causes an increase in the abundance and activity of monoderm bacteria within the microbiome (i.e., monoderm bacteria have a single unit lipid membrane and are typically Gram-positive bacteria). These shifts in activity are associated with increased activity of ABC transporter genes [74]. (iv) The extremes of temperature that occur as a consequence of climate change have been demonstrated to affect the phyllosphere and rhizosphere microbiomes of several plants [36]. (v) Low nitrogen and carbon levels, while not typically thought of as abiotic stresses, (nevertheless they may be considered to be nutrient stress) impact the soil microbiome. Thus, the presence of nitrogen-fixing trees can promote changes to the local microbiome through changes to the soil pH and C:N ratios [111]. (vi) Given how widely the herbicide glyphosate has been used in the environment for weed control, a two-year field study was undertaken to compare the soil microbiomes of plants treated with a foliar spray of glyphosate to that established in plants not treated with this herbicide [42]. Surprisingly, the microbial community of plants that were treated with glyphosate did not differ to any significant extent from the control untreated plants. (vii) Five near isogenic lines of sorghum were compared before and after a late-season frost in terms of their production of root chemicals and their rhizosphere microbiomes [62]. It was observed that the compound luteolinidin (a 3-deoxyanthocyanidin) increased in three of the lines, while flavonoids decreased in all five lines following the frost. Moreover, the composition of the rhizosphere communities of these five lines changed in concert with the changes in luteolinidin and flavonoids. Thus, some freezing affects the synthesis of some plant secondary metabolites, which in turn, affects the rhizosphere microbiome. (viii) While it has been well established that individual bacterial strains can facilitate the nodulation of legumes by various rhizobial strains [112], recent experiments have demonstrated that under nitrogen limiting conditions in the environment, microbial rhizosphere communities can associate with rhizobia and improve the nodulation of their host legumes [113]. (ix) Although it is difficult to pinpoint a particular abiotic stress, it has been observed that the pressure of urbanization can alter the rhizosphere microbiomes of beech and poplar trees [56].

The studies that have examined changes of the plant microbiomes as a consequence of abiotic stress, although mostly preliminary and incomplete, suggest that when plant growth is disturbed to a significant extent, the plant microbiome also is altered. The plant microbiome may, in some instances, protect the plant from the deleterious effects of abiotic stress [114]. Nevertheless, it is likely that different plant microbiomes will respond in different ways to the wide range and intensity of abiotic stresses.

## 4. Artificial Seed Microbiome

The bulk of microbiome research has focused on elaborating the identity of the large number of bacteria and fungi that are present in the plant rhizosphere of various plants. However, since it has become clear that the judicious use of these microbiomes may hold the key to reproducibly promoting plant growth in the field, to study and understand seed microbiomes has become more and more important. Although the number of studies of seed microbiomes is relatively limited [75,115,116,117,118,119,120], there is a very real prospect that seed microbiomes have the ability to be transmitted to, and therefore benefit, the next generation of plants. While bacterial and fungal microbiomes may be present both on the seed surface or endophytically within the seed, it is only the endophytic organisms that can be reliably transmitted to the next generation of plants. 

The bacteria that are often found associated with plant seeds include a relatively limited range of species, typically represented by members of the phyla Proteobacteria, Actinobacteria, Firmicutes and Bacteroidetes, reflecting their dominance in many soils. However, seed endophytic microbiomes are often quite distinct from those occurring in the soil where the plants have been grown. This observation is consistent with the possibility that seed endophytic microbiomes are derived from the ‘mother’ plant.

One group of scientists developed a clever and promising technique wherein selected PGPB strains were introduced into seeds, so that the seeds no longer needed to be treated by incubation en masse to deliver the microbes to growing plants. In this technique, the seed microbiome was appended with one or two particularly effective endophytic PGPB. Following the successful demonstration that using this approach it was possible to effectively introduce a single PGPB strain into seeds, the next logical step would be to design a synthetic seed microbiome (see the next section) that efficiently facilitates optimal plant growth and development in a variety of environmental conditions. 

**Figure 3:**
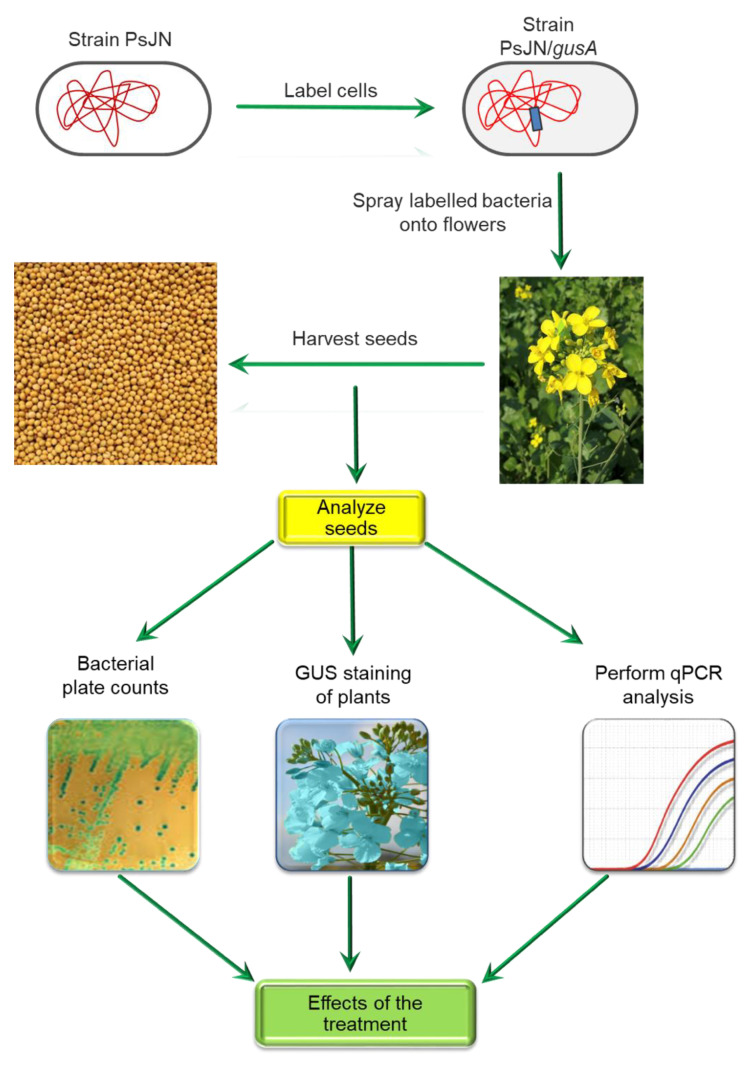
Schematic overview of a procedure used to introduce specific endophytic bacteria into plant seeds to become a component of the plant root microbiome. The gusA gene encodes the enzyme β-glucuronidase.

In a proof of principle experiment, researchers introduced the endophytic PGPB *Paraburkholderia* (formerly *Burkholderia*) *phytofirmans* PsJN [121,122] into the seed microbiomes of maize, pepper and soybean plants (Figure 3). First, the chromosomal DNA of *P*. *phytofirmans* PsJN was labeled with an *E. coli* β-glucuronidase gene, where production of this enzyme is largely confined to *E. coli* [123]. The gene encoding this enzyme is often used as a reporter gene, useful to monitor gene expression in plant and animal cells. The labeled bacteria were grown for two days to stationary phase and then a bacterial suspension of these labeled bacteria was used to spray plant flowers (corn, pepper or soy) prior to their continued growth in the greenhouse. The plants were then grown to maturity and the seeds that they produced were harvested. Following storage for 2–7 months, the newly produced seeds were tested for the presence of β -glucuronidase-labeled *P. phytofirmans* PsJN. These tests included plate counts of bacteria associated with seed extracts, staining of seed tissues for the presence of a functional β -glucuronidase enzyme (that turns the tissue blue in the presence of a specific substrate), qPCR to detect the presence of *P. phytofirmans* PsJN genes within the seeds, and detailed assessment of the altered physiology and biochemistry of the mature plants that were produced from the harvested seeds. In these experiments, it was ascertained that the efficiency of introducing strain PsJN into seeds was as high as 90%, although this varied with the different plants and the precise conditions that were employed. Importantly, this proof-of-concept experiment demonstrates that it is possible to efficiently introduce targeted PGPB strains to the seed microbiome (Figure 3).

Unfortunately, for this approach to be used on a large scale, e.g., in the development of organic agriculture, it may need to be optimized for each individual plant species. Moreover, it was observed that *P. phytofirmans* PsJN was not found in the next generation of seeds, thereby necessitating that this procedure be repeated for each plant generation. Alternatively, it is necessary to test whether strains other than *P. phytofirmans* PsJN persist in the plant microbiome for more than a single generation and to define those traits that are responsible for that persistence. In addition, as scientists develop a better understanding of microbiome interactions, it may be possible to introduce entire synthetic microbiomes into seeds, rather than a single PGPB, using this procedure. 

## 5. Synthetic Microbiomes

The first commercialized microbial inoculants generally included a single bacterium or fungus [124]. More recently, several commercial strains have been applied simultaneously [125,126]. According to Santoyo et al. [88], three different types of microbial consortia may be envisioned: those that include (i) several bacterial strains [112,127,128,129], (ii) consortia including bacteria and mycorrhizae [104], and (iii) consortia containing both bacteria and plant growth-promoting fungi (such as *Trichoderma* spp.) [130,131]. To date, the largest number of successful microbial consortia contain just two microorganisms that do not inhibit one another’s growth or functioning. For effective consortia, it is imperative that consortia members positively interact with each other over a prolonged period of time [132]. In this regard, a few successful consortia containing more than two microorganisms have been reported. These include: (i) *Xanthomonas* sp., *Stenotrophomonas* sp., and *Microbacterium* sp. [133], (ii) *Bacillus cereus* Y5, *Bacillus* sp. Y14, and *Bacillus subtilis* Y16 [134], (iii) *Brevibacillus fluminis*, *Brevibacillus agri*, and *Bacillus paralicheniformis* [135] (iv) *Bacillus cereus* AR156, *Bacillus subtilis* SM21, and *Serratia* sp. XY21 [136], and (v) *Ochrobactrum pseudogrignonense* RJ12, *Pseudomonas* sp. RJ15, and *Bacillus subtilis* RJ46 [137].

Naturally occurring microbiomes typically contain hundreds to thousands of different microorganisms. Thus, we are a long way from being able to synthetically construct such complex consortia. However, within the next 5–10 years, it should be possible to define and test effective consortia with a small number of different microorganisms. These consortia could then be added to existing seed microbiomes [121] in an effort to develop stable semi-synthetic microbiomes that are able to impart defined beneficial properties to growing plants.

Although conceptually quite different from assembling synthetic microbiomes from known and well-studied bacteria, it is possible to add specific chemicals to the soil and thereby increase the numbers of certain bacterial strains while decreasing the numbers of other strains [53,58,62,68,69,138,139]. This reflects the fact that plant metabolites often have a large impact on the bacterial community in the soil. For example, in one recent set of experiments, three different plant metabolites: benzoxazolinone, gramine and quercetin were added to agricultural soil over a period of 28 days [139]. During this period of time, bacterial diversity was significantly reduced by both benzoxazolinone and quercetin, but not by gramine. Overall, the effects of adding these compounds were characteristic for each of these three compounds. The effect of adding benzoxazolinone was predominantly inhibitory with only a few genera able to proliferate. Conversely, gramine and quercetin caused the proliferation of many plant beneficial bacterial strains. Consequently, plants that produce one or more of these metabolites should have a specific effect on the soil bacterial community. Since a large number of plant-synthesized metabolites are likely to affect the proliferation of the soil bacterial community, to specifically structure this bacterial community, it is first necessary to document the effect of various plant metabolites on the soil biota before attempting to tailor plant bacterial microbiomes by adding one or more metabolites to the soil.

## 6. Microbiomes of Transgenic Plants

Literature reports of studies of the microbiomes of transgenic plants are currently quite limited. However, this situation is likely to change in the next few years. More than 150 different plant species have already been genetically transformed and by 2018 more than 20 million farmers in 26 different countries worldwide were using this technology with major transgenic crops including soybean, corn, cotton, canola, alfalfa, rice, squash, papaya, wheat, eggplant, potatoes, sugarcane and apples [140]. While the biochemistry and physiology of a transgenic plant generally does not change to any appreciable extent, sometimes even different cultivars of the same plant have been observed to differ in the composition of their root exudates and rhizosphere/endophere microbiota [41]. Thus, it is important to characterize the root exudates and microbiomes of transgenic plants to ensure that, where possible, we are able to understand and optimize the plant microbiomes of the transgenic plants to the same extent as their non-transgenic counterparts. 

To date, scientists have reported the following. (i) When transgenic switchgrass plants that had been engineered to contain a decreased lignin content were grown in the field, over a period of two to five years, there was no effect of the transgenic plants on rhizospheric bacterial diversity, richness, or community composition [141]. (ii) In another study, rice plants were transformed with a Cry1Ab/1Ac gene which encodes a *Bacillus thuringiensis* insecticidal protoxin yielding a plant that is similar to other transgenic plants that have previously been released into the environment [31]. In this case, the transgenic rice did not confer any significant effect on the soil bacterial community structure. Again, this result suggests that generation of transgenic rice did not have a significant effect on the plant rhizosphere microbiome. (iii) A study of sugarcane plants that had been genetically engineered to overexpress the *Ea-DREB2B* gene in an effort to increase the drought tolerance of these plants revealed that the rhizosphere bacterial community of the transgenic plants were changed in response to changes in the plant root exudates [67]. From these limited studies, the preliminary conclusion may be drawn that transgenic plants do not have significantly altered root exudates and rhizosphere bacterial microbiomes unless the introduced transgene alters the behavior of the plant in the natural environment.

## 7. Conclusions

A current, but not new, concept describes the plant and its associated microbiome as a complex multi-organ entity called the “holobiont”. Although the term holobiont was originally proposed by Adolf Meyer-Abich (for a historical essay see the review by Amidon 2009) [142], it is most often known as associated to Lynn Margulis who, in 1991 [143], formulated the endosymbiotic theory. The vision of the plant as an holobiont is represented by an incredibly intricate net of interactions connecting the plant host with its endocellular and extracellular microbiome and, of course all the members of the microbiota to each other. In such a complex web of relationships, each variation in the microbiome determines shifts in the net of interactions amongst all the organisms involved [144]. However, the recent concept of the core microbiome, highlighted the occurrence of “sets of microorganisms that form cores of interactions that can be used to optimize microbial functions at the individual plant and ecosystem levels” [145]. Typically, the core microbiome includes microorganisms that are stably associated with one plant species, irrespective of the soil physical and chemical characteristics and of the environmental conditions. While the core microbiota, at taxonomic level, is the key driving the organization of the plant microbiome, the functional core microbiota is composed by those microorganisms which are able to provide key functions for the maintenance of the holobiont fitness through nutrition and health improvement [146]. In this context of coevolution, PGPB and plants have interacted and facilitated one another’s growth for around 50 million years. Thus, although most PGPB and plants are able to grow and proliferate on their own, the growth and persistence of both partners benefit significantly under natural environmental conditions when PGPB and plants work together; this is especially true during periods of environmental stress [114]. Unfortunately, a single PGPB strain, no matter how effective it might be in facilitating plant growth under controlled laboratory conditions, is extremely unlikely to be able to meet all of a plant’s needs in the natural (and potentially changing) environment. Conversely, groups of microorganisms (predominantly bacteria and fungi), working cooperatively, each with somewhat different metabolic features, are better suited to facilitate plant growth (and possibly the growth of their microbiome partners as well) under a very wide range of natural environmental conditions [147]. As is often the case for human interactions, the group is generally stronger and more effective than the sum of its parts.

As scientists continue to develop an increased understanding of the mechanisms employed by individual PGPB to facilitate plant growth and development, these bacterial strains will be used to a much greater extent in plant agriculture replacing many of the chemicals that are currently employed. For the use of PGPB to be even more effective in the future, it will be necessary to utilize beneficial consortia of microorganisms acting as directed microbiomes able to promote the growth of target plants under a wide range of environmental conditions. In an era where climate change is dramatically impacting the natural world, plants are not necessarily limited in their ability to thrive once the climate has changed. Rather plant success may become even more dependent upon plant interactions with specific microbiomes. Considering both the relative infancy of the field of plant microbiomes as well as its rapid pace of progress, it is not unrealistic to expect some of these types of consortia to be commercially available within the next 5–10 years.

## Figures and Tables

**Table 1:** The microbiomes of various plants.

Plant	Location	Comment	Reference
*Artemisia herba-alba*	Rhizosphere	Highest number of bacterial species compared to the microbiota of 13 other plant species of the Algerian desert.	[25]
*Brassica napus* (canola)	Rhizosphere	Examined field grown plants at 3 separate sites. Found stable bacterial core microbiome.	[26]
*Brassica napus*	Rhizosphere	Different microbiome complexity at different stages of plant growth.	[27]
*Oryza sativa* (rice)	Root surface, root endosphere, shoot surface, shoot endosphere	Compared microbiome of rice seedlings to microbiome of rice seed. Greatest abundance and diversity found in roots.	[28]
*Oryza sativa*	Rhizosphere, endosphere, rhizoplane	The three root-associated compartments that were studied, each had distinct microbiota.	[29]
*Oryza sativa*	Rhizosphere	Elevated levels of CO_2_ suppresses methane oxidation thereby promoting methanogenesis in rice roots.	[30]
*Oryza sativa*	Rhizosphere soil, plant stems/leaves, plant roots	Transgenic rice expressing a Bt protoxin gene did not significantly change the plant bacterial strains compared to the parental strain.	[31]
*Oryza sativa*	Rhizosphere	*Indica* and *japonica* varieties recruit distinct root microbiota. *NRT1.1B*, a rice nitrate transporter, is involved in recruitment of the *indica*-enriched bacteria.	[32]
*Oryza sativa*	Endosphere	In three different varieties, the endophytic microbiome varied significantly between young and mature plants.	[24]
*Vitis vinifera* (grape)	Rhizosphere	Compared rhizosphere to bulk soil in a conventionally managed vineyard.	[33]
*Vitis vinifera*	Rhizosphere	Microbiomes of the same cultivar were different when they were grafted onto 2 different rootstocks.	[34]
*Vitis vinifera*	Rhizosphere	Compared rhizosphere to bulk soil in an integrated pest management vineyard.	[35]
*Rubus chamaemorus, Andromeda polifolia, Empetrum vaginatum, Sphagnum* sp., *Carex rotundata, E. angustifolium*	Phyllosphere and rhizosphere	All plants were from arctic peatlands. Microbiomes were compared to peat. Methanogen abundance was strongly influenced by the individual plant.	[36]
*Zea mays* (corn)	Rhizosphere	Samples were from corn farms.	[37]
*Zea mays*	Rhizosphere	Isolated and sequenced the genomes of several rhizosphere bacteria.	[38]
*Zea mays*	Endosphere	Strong relationship between endosphere community and corn productivity.	[39]
*Zea mays*	Rhizosphere	The rhizosphere community following crop rotation was more abundant than following monocropping.	[40]
*Zea mays*	Bulk soil, rhizosphere, endosphere	Different cultivars had different biomass, root exudates and different microbiota in bulk soil, rhizosphere and endosphere. Also, different soils contributed to microbiome variation.	[41]
*Zea mays* and *Glycine max* (soybean)	Rhizosphere	Found no significant difference between plants treated with glyphosate and those not treated with this herbicide.	[42]
*Glycine max*	Rhizosphere	Determined the effect of nodulation phenotypes on soybean microbiomes.	[43]
*Brassica napus*, *Buglossoides arvensis* (corn gromwell) and *Glycine max*	Rhizosphere	Inoculation with *Pseudomonas* strain promoted seed oil accumulation, increased abundance of 29 taxa and decreased abundance of 30 taxa.	[44]
*Gossypium hirsutum* (cotton)	Rhizosphere	Characterized the microbiome associated with *Verticillium* wilt.	[45]
*Gossypium hirsutum*	Rhizosphere, bulk soil	Biota diversity increased in soil with cotton plants. Drought stress increased the abundance of some bacteria which help sustain the plants.	[46]
*Triticum aestivum* (wheat)	Rhizosphere	Compared eight wheat cultivars grown under field conditions for root diameter and root length and microbiome.	[47]
*Triticum aestivum*	Rhizosphere	Irrigation adversely affected the bacteria that produce the antibiotic phenazine-1-carboxylic acid.	[48]
*Triticum aestivum*	Rhizosphere	Examined effect of long term nitrogen fertilization. *Acidobacteria* increased and *Actinobacteria* decreased.	[49]
*Fragaria* x *ananassa* (strawberry)	Rhizosphere	Examined 16 strawberry cultivars in two field studies. Plants had a genotype-dependent microbiome.	[50]
*Curcurbita pepo* (pumpkin)	Rhizosphere, seed and soil	Seed microbiome diversity is lower than rhizosphere or soil.	[51]
*Solanum tuberosum* (potato)	Tuber microbiome	Examined four potato varieties and five soil types. In all cases, bacterial community shifted from harvest to dormancy break.	[52]
*Ipomoea batatas* (sweet potato)	Rhizosphere	Adding low level of urea to soil increased abundance of P- and K-solubilizing bacteria, and N-fixing bacteria.	[53]
*Populus cathayana* (poplar)	Phyllosphere	Both female and male plants had unique bacterial microbiota.	[54]
*Picea* spp. (spruce)	Rhizosphere, phyllosphere	Correlations between microbiota and plant phenotypes suggest that plant genotype determines microbiota.	[16]
*Populus trichocarpa* (black cottonwood)	Phyllosphere endosphere	Observed a core microbiome. Nevertheless, variation existed between trees growing at different sites.	[55]
*Fagus grandifolia* (beech), *Liriodendron tulipifera* (yellow poplar)	Soil surrounding tree, rhizosphere	Soil microbial communities are unique to each tree species, however, urbanization decreased these differences.	[56]
*Solanum lycopersicum, S. pimpinellifolium* (tomato)	Rhizosphere and root endosphere	Examined eight tomato varieties and found that both endosphere and rhizosphere were affected by plant genotype.	[57]
*Solanum lycopersicum*	Rhizosphere	In tomato plants, the rhizosphere microbiota in neighboring plants is affected by volatile organic compounds.	[58]
*Thalassia hemprichii, Enhalus acoroides* (tropical seagrass)	Rhizosphere	This data suggests that the main determinant in selecting the rhizosphere microbiome is the plant habitat and not the plant species.	[59]
*Persea americana* (avocado)	Rhizosphere	Phytophthora root rot modified the bacterial composition and increases the amount of opportunistic fungal pathogens.	[60]
*Pisum sativum* (pea)	Seeds	Compared microbiota of seeds from 3 different countries. All peas shared a common core microbiota but also showed differences according to origin.	[61]
*Sorghum bicolor* (sorghum)	Rhizosphere	Microbiota of 5 different lines of sorghum were correlated with total flavonoid and luteolinidin concentrations.	[62]
*Sorghum bicolor*	Rhizosphere	Drought significantly delays the development of the root microbiome.	[63]
*Panicum virgatum* (switchgrass)	Shoots, roots and root-influenced soil	Different plant parts have different microbiomes (which are also influenced by climate, season and host genotype).	[64]
*Panicum virgatum*	Rhizosphere	Each of 12 cultivars that were tested selected a different microbiome.	[65]
Legumes	Nodules	Highly diverse population of bacteria within nodules that do not elicit nodulation or nitrogen fixation.	[66]
*Saccharum arundinaceum* (sugarcane)	Rhizosphere, rhizoplane, bulk soil	Bacterial communities of the transgenic plants were altered in comparison to the wild-type plant communities.	[67]
*Arabidopsis thaliana*	Rhizosphere	Coumarin biosynthesis dictates root biota composition.	[68]
*Arabidopsis thaliana*	Rhizosphere	Three different root triterpenes dictate root biota composition.	[69]
*Arabidopsis thaliana*	Rhizosphere	The defense hormone salicylic acid modulates root colonization by specific bacteria.	[70]
*Arabidopsis thaliana*	Rhizosphere	Used synthetic microbiome. Found *Variovorax* spp. responsible for optimizing root growth.	[71]
*Arabidopsis thaliana*	Phyllosphere	Isolated and sequenced 275 microbiomes. Found only weak associations with site of origin and plant genotype.	[72]
*Oxyria digyna, Saxifraga oppositifolia*	Endosphere	The plants shared a core microbiome. In addition, geographic region was a major determinant of biota composition.	[73]
Citrus	Rhizosphere	Characterized rhizospheres and bulk soil from 23 locations worldwide including 7 soil types and 6 climate types and 12 plant varieties and found a core microbiome.	[74]
Apples	Fruit	Different tissues, including stem, peel, fruit pulp, seeds and calyx, had distinct bacterial microbiomes.	[75]
*Echinacea purpurea* (purple coneflower), *E. angustifolia*	Rhizosphere, stem, leaf	Bacterial microbiomes were significantly different in these two plants and within different tissues.	[14]
*Phoenix dactylifera* (date palm)	Root endosphere	Bacterial and fungal community structures were not significantly affected in the presence of high salt.	[76]
*Phoenix dactylifera*	Root and leaf endosphere	Leaf and root tissues respond differently to salt stress.	[77]
*Medicago truncatula* (caliph medic)	Root endosphere	The abundance of ~70% of the biota characterized was altered in the presence of high salt.	[78]
*Cucumis sativus* (cucumber)	Rhizosphere	A *Bacillus amyloliquefaciens* strain addition significantly altered the bacterial rhizosphere community.	[79]
*Hordeum vulgare* (barley)	Rhizosphere	Comparing wild-type and root hair mutant barley, root hairs are critical in determining rhizosphere community.	[18]
